# Efficacy of Dronabinol for Acute Pain Management in Adults with Traumatic Injury: Study Protocol of A Randomized Controlled Trial

**DOI:** 10.3390/brainsci10030161

**Published:** 2020-03-12

**Authors:** Claire Swartwood, Kristin Salottolo, Robert Madayag, David Bar-Or

**Affiliations:** 1 Pharmacy Department, St Anthony Hospital, Lakewood, CO 80228, USA; Claireswartwood@centura.org; 2 Trauma Research Department, St Anthony Hospital, Lakewood, CO 80228, USA; Kristin.salottolo@icloud.com (K.S.); robertmadayag@centura.org (R.M.); 3 Trauma Services Department, St Anthony Hospital, Lakewood, CO 80228, USA

**Keywords:** Delta-9-tetrahydrocannabinol, dronabinol, marijuana, randomized controlled trial, opioids, traumatic injury

## Abstract

Delta-9-tetrahydrocannabinol (Δ^9^-THC) and other cannabinoids present in cannabis (marijuana) have been shown to affect the normal inhibitory pathways that influence nociception in humans. The potential benefits of cannabinoids as an analgesic are likely greatest in hyperalgesic and inflammatory states, suggesting a role as a therapeutic agent for treating acute pain following injury. Dronabinol is a licensed form of Δ^9^-THC. The primary objective of this single center randomized controlled trial is to evaluate the efficacy of adjunctive dronabinol versus control (systemic analgesics only, no dronabinol) for reducing opioid consumption in adults with traumatic injury. Study inclusion is based on high baseline utilization of opioids ≥50 morphine equivalents (mg) within 24 h of admission for adults aged 18–65 years with traumatic injury. There is a 48-hour screening period followed by a 48-hour treatment period after randomization. A total of 122 patients will be randomized 1:1 across 2 study arms: adjunctive dronabinol versus control (standard of care using systemic analgesics, no adjunctive dronabinol). Patients randomized to the dronabinol arm should receive their first dose within 12 h of randomization, with a dose range of 5 mg up to 30 mg daily in divided doses, in addition to systemic analgesics as needed for pain. The primary efficacy endpoint is a change in opioid consumption (morphine equivalents), assessed post-randomization (48 h after randomization) minus pre-randomization (24 h prior to randomization). This is the first randomized trial to investigate whether adjunctive dronabinol is effective in reducing opioid consumption in acute pain management of traumatic injury. Trial Registration: ClinicalTrials.gov Identifier: NCT03928015.

## 1. Introduction

Delta-9-tetrahydrocannabinol (Δ^9^-THC) and other cannabinoids present in cannabis (marijuana) have been shown to affect the normal inhibitory pathways that influence nociception in humans. Cannabinoids act through the binding of two cannabinoid receptors coupled through G proteins; CB1 receptors are predominantly found at central and peripheral nerve terminals, where they mediate transmitter release, while CB2 receptors are highly expressed throughout the immune system [1].

The evidence demonstrating a therapeutic effect of THC and cannabis-based medications is still emerging but is well established for treating chronic pain based on three influential peer-reviewed publications [2,3,4]. These publications also provide conclusive evidence for a therapeutic effect of cannabis-based medications as anti-emetics and for multiple sclerosis symptoms. There is moderate evidence for improving sleep outcomes associated with sleep apnea, fibromyalgia, multiple sclerosis, and chronic pain. There is insufficient or low-quality evidence in all remaining conditions that have been studied. For instance, there is a dearth of research on cannabinoid use for acute pain management. A 2017 systematic review identified seven randomized controlled trials (RCT) assessing the analgesic efficacy of cannabinoid medications for acute pain [5]. Of these studies, five RCTs demonstrated that cannabinoids were equivalent to placebo, in one RCT cannabinoids were superior to placebo, and in one RCT cannabinoids were inferior to placebo. These limited and inconsistent data justify the necessity to perform additional studies on the analgesic effects of cannabinoids for acutely painful conditions.

Patients commonly experience severe, acute pain following traumatic injury that is treated with analgesics, particularly opiates. The antinociceptive properties of cannabinoids may be greatest in hyperalgesic and inflammatory states, suggesting a therapeutic role for treating pain following injury [6]. Moreover, pre-clinical studies support a potential role of Δ^9^-THC and cannabinoids as an adjunctive agent to opioids in painful conditions, via synergistic enhancement of mu opioid antinociception as well as the prevention of tolerance to and withdrawal from opiates [7,8,9].

Recently published preliminary clinical research from our group examined the effect of adjunctive dronabinol for acute pain management among 66 trauma patients [10]. Cases demonstrated a significant reduction in opioid consumption (morphine equivalents) from baseline with adjunctive dronabinol (−79 mg, *p* < 0.001), while the change in opioid consumption for matched controls was unchanged from baseline (−9 mg, *p* = 0.63), resulting in a nine-fold greater reduction in opioid consumption for cases versus controls that was significantly different between pairs (difference: −70 mg, *p* = 0.02). There were no differences in secondary outcomes. These results suggest that adjunctive dronabinol used as part of a multimodal analgesia regimen may result in a marked reduction in opioid consumption

Two subset analyses of this matched cohort study provide mixed evidence that the opioid sparing effect of dronabinol may be greater in patients who are marijuana users. Among the subset of 19 cases who were marijuana users, opioid consumption was significantly reduced with adjunctive dronabinol (−97 mg, *p* < 0.001) versus no change in opioid consumption in 19 matched controls (1 mg, *p* = 0.70), with a difference between pairs that was significant: −108 mg, *p* = 0.01) [10]. However, when examining the subset of patients who received dronabinol, there were no differences in the change in opioid consumption for patients who were marijuana users (*n* = 21, −97 mg reduction with dronabinol) compared to non-marijuana users (*n* = 15, −64 mg reduction with dronabinol), *p* = 0.41 (unpublished). 

We are recruiting patients in a RCT to evaluate the efficacy of adjunctive dronabinol on opioid utilization for acute pain management. The primary trial objective is to evaluate the efficacy of adjunctive dronabinol versus control (systemic analgesics only, no dronabinol) for reduction in opioid consumption in adults with traumatic injury. Dronabinol is a licensed form of Δ^9^-THC. Dronabinol is not FDA approved for acute pain management; however, it has been in use at our level I trauma center system formulary without restriction since 2015. 

## 2. Materials and Methods

### 2.1. Study Design and Setting 

This is an open label RCT being performed at a single level I trauma center: St. Anthony Hospital in Lakewood, CO. This RCT was designed primarily to determine whether adjunctive dronabinol reduces opioid consumption compared to control. The study was designed with a stratified randomization by baseline marijuana use, which is intended to determine whether the treatment effect of dronabinol is greater in chronic marijuana users compared to recreational or non-marijuana users. This stratified randomization design was incorporated based on the gestalt that cannabis-based medication has a greater benefit for marijuana users.

There is a 48-hour screening/randomization window, a 48-hour treatment window, and a total participation period extending through the acute hospitalization. A description of the clinical trial is posted at ClinicalTrials.gov.

### 2.2. Study Subjects

**Figure 1:**
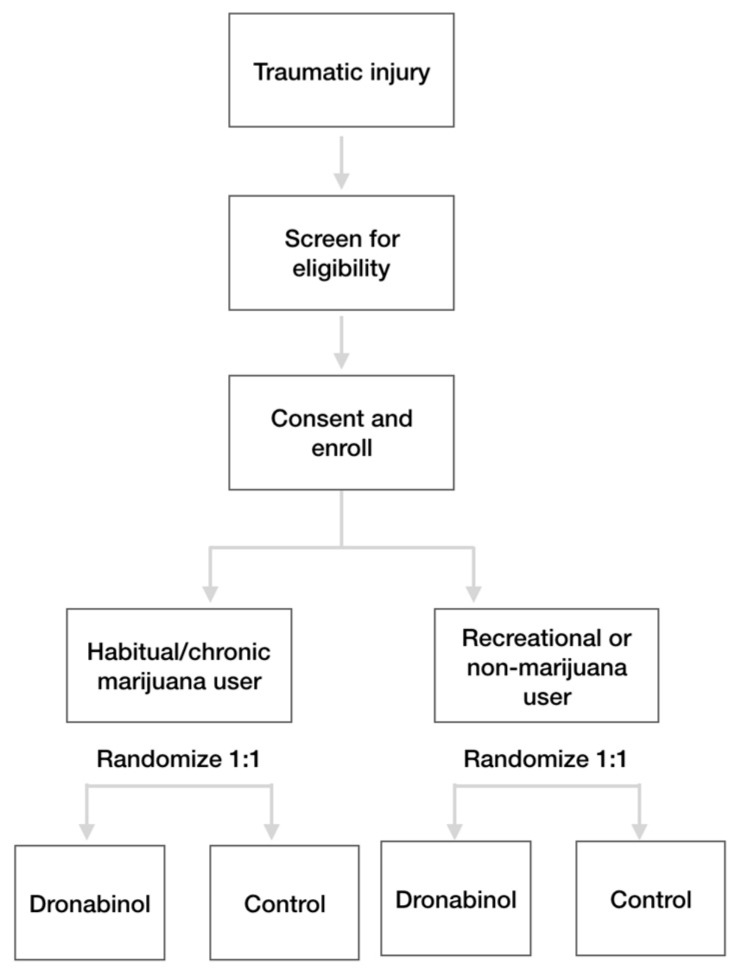
Subject Disposition.

Patients are being recruited from the participating trauma center to which they are acutely presenting. A total of 122 adult trauma patients will be randomized 1:1 across 2 study arms: adjunctive dronabinol or control (systemic analgesics only), as shown in Figure 1. 

Patients should fulfill all of the following inclusion criteria:

- Male or female, 18 years to 65 years old (inclusive).
- Diagnosis of traumatic injury based on ICD10 diagnosis of S00-T14, which covers injuries to any region of the body.
- Moderately high initial morphine equivalent use ≥50 mg within a 24-hour window during the screening period. Opioids will be converted to morphine equivalents (mg) using an Equianalgesic conversion chart, Table 1 [11].
- Willing to disclose current marijuana status (current user (habitual/chronic or recreational), former user, never user).

Patients fulfilling one or more of the following criteria may not be enrolled in the study:

- Patients on a pain management agreement
- Patients who are nil per os (NPO) at the time of randomization or are expected to be NPO within the next 48 h, with the exception for a brief NPO period for surgical procedures
- Patients who have received or are expected to receive neuraxial/locoregional blocks for pain within the next 48 hours
- Patients with a known allergy or previous hypersensitivity reaction to dronabinol or sesame oil
- Patients prescribed dronabinol between arrival and prior to randomization
- Pregnancy or breast feeding
- Incarceration (presumed; patients are not arraigned until after hospital discharge).

### 2.3. Study Visits

The following procedures will be performed at screening, within 48 h of hospital admission: ensure patient meets inclusion and exclusion criteria; record 24-hour total morphine equivalents; record habitual marijuana usage; obtain informed consent via patient or proxy. 

Once patients are confirmed to meet all criteria and have signed an informed consent, they will be randomized 1:1 across the two study arms (dronabinol or control, Figure 1). The following procedures will be performed during randomization: randomize the patient using the Microsoft Excel blinded randomization schema; record pain using the patient self-reported pain numeric rating scale (NRS, 0–10 scale). 

The following procedures will be performed during the acute hospitalization, post-randomization: if randomized to the dronabinol arm, administer the first dose within the first scheduled dose window and within 12 h of randomization. Record all doses received, including date, time and dose; record all opioid and non-opioid systemic analgesics received, including route/dose/frequency; record all non-analgesic concomitant medications; record pain NRS scores at the following time points: once admitted in hospital bed, preoperatively in the OR prior to anesthesia, one hour post operatively; record all analgesic complications; record all documented drug use from patient self-report and urine drug screening results. Detailed information on the regularity of marijuana use will also be recorded. 

The following procedures will be performed at hospital discharge: record discharge pain NRS score; record discharge location; re-consent, if necessary. 

### 2.4. Randomization and Blinding

Patients will be assigned to treatment by a randomization schedule developed and maintained by an independent statistician. The randomization allocation sequence was computer generated and is blinded, with allocation hidden until a patient has met all inclusion and exclusion criteria and provided informed consent. Randomization will occur in a 1:1 fashion in blocks of 2 and 4 and stratified by habitual marijuana user (yes/no). 

The assessor, participants, treatment team, and statisticians are unblinded. All assessments are standard and routinely collected by the assessors (ICU and general ward nursing staff), including pain NRS scores and analgesia administration. 

### 2.5. Intervention

The study drug is dronabinol (Marinol®, AbbVie, Inc; Chicago, IL, USA). Eligible patients will receive adjunctive dronabinol vs. control (no dronabinol, systemic analgesia only). Patients will be allocated to a treatment in accordance with the randomization schedule following confirmation of eligibility. 

Patients who consent to participate in the study will have an order in the electronic medical record that will be used to assist with treatment compliance and for dispensing dronabinol, when applicable. Patients randomized to the dronabinol arm should receive their first dose within 12 h of randomization. The initial dosing and any changes in dosing will be determined by the prescribing/treating clinician. The target dose is 5 mg twice daily; the dose may be adjusted to within 5 mg to 30 mg daily in divided doses (e.g., 2.5 mg twice daily–10 mg three times daily). Patients who are randomized to the control arm will have an order set that specifies no administration of dronabinol for 48 h.

Patients in both arms will receive as needed (pro re nata, PRN) non-opioid/opioid analgesia as determined by the care team; patients who are not randomized to the dronabinol arm will receive these analgesics only, while patients randomized to the dronabinol arm will receive dronabinol in addition to PRN non-opioid/opioid analgesia. A target pain numeric rating score for trauma patients is 4 or less on a 0–10 scale. Higher pain scores ≥5 typically warrant analgesia, as determined by the attending physician and care team for the patient’s specific needs. These established guidelines will ensure patients are receiving analgesia based on self-reported pain, independent of treatment arm.

After the 48-hour treatment window post-randomization, the use of adjunctive dronabinol for the remaining acute hospitalization will be at the patient’s and physician’s discretion. Except for the analgesia protocol, all other interventions will follow techniques used in the context of everyday clinical practice, and thus will be identical for participants in both arms. The following medications are discouraged: neuraxial and locoregional nerve blocks.

### 2.6. Outcome Measures

Patients will be followed to hospital discharge for outcomes of morphine equivalent use, length of stay, pain NRS scores, hospital complications, and analgesic complications. 

The primary outcome is morphine equivalents. All opioids consumed will be converted to morphine equivalents, as shown in Figure 1 [11]. The clinical effects of treatment arm on morphine equivalents will be evaluated at 48 h after randomization. 

Secondary outcomes include the following:

- Morphine equivalents: overall (hospital admission through discharge or death)
- Non-opioid analgesics: overall doses received (admission through acute hospitalization discharge or death), and examined by non-opioid drug
- Acute hospitalization length of stay
- Pain NRS scores: in ED prior to randomization, once admitted in hospital bed, preoperatively in the OR prior to anesthesia, one-hour post operatively, at hospital discharge
- Time (hours) to transition to non-opioid analgesia
- Incidence of hospital complications
- Safety (Incidence of analgesic complications)

Analgesic complications will be recorded irrespective of the presence or absence of a causal relationship, and include the including:

- Allergic reaction
- Nausea and vomiting
- Respiratory depression (hypoxia and hypopnea)
- Hypotension
- Urinary retention
- Constipation/ileus
- Abdominal pain
- Dizziness
- Euphoria
- Paranoid reaction
- Somnolence
- Delirium
- Over-sedation.

### 2.7. Statistics

Significance is set at an alpha value of 0.05. SAS (Cary, NC) software will be used for statistical analysis. All efficacy analyses will be performed in the intent-to-treat population, defined as all patients who are randomized. Subset analyses will be performed by habitual marijuana use. 

The primary endpoint is the change in morphine equivalents and will be assessed as: post-randomization (48 h after randomization) minus pre-randomization (24 h prior to randomization). No imputation will occur for the primary endpoint. The change in morphine equivalents (mg) will be analyzed with an analysis of covariance (ANCOVA) model to examine the effect of treatment arm, adjusted for age, gender, injury severity score, and clinical characteristics that differ between groups with *p* < 0.15. Of note, our study inclusion criteria allow for patients to present with polytrauma. We anticipate the majority of patients will have injuries to the thorax and extremities, with few patients presenting with severe TBI because administration of opioids and other drugs that alter a neurological assessment tend to be used sparingly. Should there be differences in injury patterns, despite the 1:1 randomization procedure, these differences will be adjusted for in the primary ANCOVA analysis.

Secondary efficacy analyses include the difference between treatment groups in: hospital disposition, hospital complications, and analgesic complications, reported as proportion (%) and analyzed with chi-square tests; morphine equivalents over the hospitalization, hospital length of stay (days), time (h) to transition to non-opioid analgesia, pain NRS scores at all specified time points, reported as median (IQR) and analyzed with a Wilcoxon rank-sum test. Analgesic complications will be described by severity as mild, moderate and severe.

### 2.8. Sample Size

The planned enrollment is 122 patients total randomized 1:1 across two study arms: dronabinol or no dronabinol (systemic analgesia only). The sample size is based on a 38% reduction in morphine equivalents with adjunctive dronabinol vs. an 8% reduction in morphine equivalents for systemic analgesics only, with a pooled standard deviation of 58. The analysis was performed using two sample mean tests with normal approximation and equal weights. These estimates were derived via bootstrapping of the final matched study sample of 66 patients. The power to demonstrate the main effect of dronabinol over systemic analgesics is 80% using a 2-tailed alpha of 0.05. 

### 2.9. Ethical Considerations

The study was approved from the Institutional Review Board for St. Anthony Hospital (Catholic Health Initiatives). There may be patients incurring cognitive impairment (due to head injury or acuity of illness). The study coordinator will discuss with the treating team and will directly assess the consenting capacity of the patient. The study nurse follows the current hospital protocol regarding the use of consent by a legally authorized representative. In these clinical situations where the patient’s representative initially consents, the patient will be "re-consented" when able to assure that they want to continue in the study. 

Safety outcomes will be reported to the head of the medical executive committee at an ongoing basis. If/when the rate and/or severity of the monitored safety events becomes unacceptable, the medical executive committee has procedures in place to protect research subjects.

An interim analysis will be performed when >50% (*n* = 62) of patients have been enrolled and discharged from the hospital to determine clinical equipoise. A stopping guideline of *p* < 0.001 will be used for the primary end point.

## 3. Discussion

This is the first randomized trial to investigate whether the addition of dronabinol is effective at reducing opioid consumption for acute pain management of traumatic injury. There are numerous strengths of this study. This clinical trial improves upon our previously published matched cohort study and removes many of the limitations of that study: patients will now be matched by self-reported marijuana use; the pre-treatment period for the controls will be identical to cases rather than being estimated based on the median time from admission to first administration of dronabinol among cases; we will know why controls were not prescribed dronabinol; there are complications and adverse effects that are associated with both systemic analgesics and dronabinol, which will be recorded and analyzed in this trial by treatment arm and by severity. Additional strengths of this study are that it is investigator-initiated and independent from pharmaceutical or other industry interests, and the findings (whether positive or negative) will be submitted to a peer reviewed scientific journal for publication.

Another benefit of this study is the stratified randomization by chronic marijuana usage. Earlier work by the study investigators suggests that pre-injury marijuana use results in increased consumption of opioid analgesics and greater self-reported pain following traumatic injury compared to trauma patients who are marijuana naïve [12]. If the randomized trial demonstrates a greater treatment effect in the subset of chronic marijuana users, this will have wide-ranging clinical implications for acute pain management, because trauma patients have a high prevalence of marijuana use and other substance abuse issues, reported in 40–50% of patients [13,14] that appears to be increasing over time [15]. Thus, if marijuana use significantly affects acute pain management then chronic marijuana users will merit special consideration during acute pain management. 

While there are now 11 states that have legalized recreational marijuana, we believe Colorado is uniquely able to study this issue because of the high utilization in our state. Colorado was the first state to legalize and commercialize recreational marijuana, with retail shops opening on January 1^st^, 2014. A recent study identified that commercialization of recreational marijuana in Colorado was associated with an increased use of marijuana or an increased risk of traumatic injury while using marijuana [15]. 

Opioids are established and effective analgesics for managing pain in the traumatic and critical care setting due to their proven efficacy in treating moderate to severe acute pain [16]. The Center for Disease Control and Prevention (CDC) estimates that approximately 130 Americans are dying each day from opioid overdose, resulting in an opioid epidemic. We believe the use of dronabinol as a tool in the clinician’s tool kit to decrease reliance on opioids is an appealing option. Some possible benefits of this study include better pain control and a lower need for opiates for participants. Use of dronabinol to reduce or maintain the opioid regimen, rather than increasing narcotic dosages to detrimentally high levels, may also reduce the negative effects of opioids on vascular neurologic response and respiratory depression.

One of the primary limitations of this trial is that the study is open label. Patients are still prospectively randomized to active treatment vs. control and all assessments are standard and routinely collected by the assessors (ICU and general ward nursing staff), including pain scores and analgesia administration. However, we are unable to blind patients because there are no orally administered placebo pills that are on hospital formulary to be used for this study (unavoidable blinding). We did not blind clinicians because the dosing of dronabinol may need to be modified and is allowed within the range of 5 mg to 30 mg daily in divided doses. Although a blinded study would be preferred to reduce knowledge bias, the study design is compatible with real-world situations and increases the external validity of the study. 

Additional limitations are as follows. First, our preliminary study was conducted in 2017, around the peak of the opioid epidemic [17,18]. Since that time, there have been enterprise-wide initiatives to use alternatives to opioids [19,20], which could impact our enrollment criteria. However, our study has potentially greater implications in the current setting where opioid alternatives are sought. Second, and related, the data used to power the RCT were recorded in 2017, and it is possible that opioid consumption will be less in both groups (dronabinol and control), but whether this translates to a different treatment effect with dronabinol remains to be seen. Third, marijuana use is based on self-reporting because admission urine toxicology testing is only utilized in about 50% of patients, with a bias towards screening younger patients. Unlike blood alcohol tests, urine toxicology testing seldom results in a change in care and thus are not routinely ordered following traumatic injury. We will not be requiring a change in practice for ordering urine toxicology testing as part of our study. However, our unpublished research demonstrates the percent agreement between urine toxicology findings and patient self-report is 81% for cannabis. The negative predictive value of 95% demonstrates that a negative self-report correctly identifies 95% of patients who test negative for cannabis, while the specificity provides an 85% chance that a patient will not test positive for cannabis if the patient denies use. Fourth, the results of this study are only be applicable to dronabinol and not to other cannabinoids, such as the recently trending cannabidiol (CBD). Finally, the study is currently approved as a single-center RCT, which limits its generalizability. The authors are amenable to adding additional sites which use dronabinol on formulary without restrictions. 

There are two additional risks to the patient that need to be mentioned. First, this study involves an experimental (investigational) drug that has not been approved by the U.S. Food and Drug Administration (FDA) for the specific indication of acute pain management. Dronabinol is only FDA approved for loss of appetite due to HIV and chemotherapy-induced nausea and vomiting. This study is not intended to result in an FDA Investigational New Drug Application. Second, dronabinol is a synthetic version of THC. There is a risk that the study medication will result in a positive urine drug screen test for cannabis for two weeks or more in patients who are not a current user of marijuana products. In most cases, if an employee has a recent prescription for dronabinol, that is sufficient to report the result to the employer as a negative. 

### Trial Status

The trial has been recruiting patients since October 2019 and will continue until 122 patients have been randomized. Protocol version 1.2. Two amendments have occurred since trial commencement. First, the inclusion criteria of a minimum baseline pain score ≥5 was removed. The second amendment modified the sample size calculation to incorporate the full preliminary study findings, rather than a smaller pilot population. 

## Figures and Tables

**Table 1:** Oral morphine milligram equivalents (MME) conversion factors.

Opioid (mg, Except Where Noted)	Oral MME Conversion Factor ^1^
Buprenorphine	N/A
Codeine	0.15
Fentanyl, intravenous (mcg)	0.3
Hydrocodone	1
Hydromorphone	4
Meperidine	0.1
Methadone	3
Morphine, oral	1
Morphine, intravenous	3
Oxycodone	1.5
Tramadol	0.1

^1^ Formula: Strength per Unit X (Number of Units/Days Supply) X MME conversion factor = MME/Day.
